# The socio-economic and cultural impacts of the Pan Borneo Highway on Indigenous and local communities in Sabah, Malaysian Borneo

**DOI:** 10.1371/journal.pone.0269890

**Published:** 2022-06-27

**Authors:** Nicola Karen Abram, Hong Ye Lim, Casey Keat-Chuan Ng, Sylvia Yorath, Mohamed Haleem Mohamed Razi, Cynthia Gaik Suan Ong, Kok-On Chen, Kenneth Brockington Wilson

**Affiliations:** 1 Forever Sabah, Kota Kinabalu, Sabah, Malaysia; 2 Land Empowerment Animals People, Kota Kinabalu, Sabah, Malaysia; 3 University College Sabah Foundation, Kota Kinabalu, Sabah, Malaysia; Universiti Malaysia Sabah, MALAYSIA

## Abstract

Road and highway development can provide multiple benefits to society, but without careful planning, this development can result in negative social and environmental impacts. The 1,200 km Pan Borneo Highway project (PBH) in Sabah, Malaysian Borneo, is constructing new highways and up-grading 2-lane roads to 4-lane highways. We assessed the potential impact of the PBH on communities using three width scenarios of 50m, 75m and 100m for planned highway alignments, and identified potentially impacted dwellings and community lands. We estimated that 65–93 villages will be impacted, and that 1,712–7,093 dwellings and 3,420–6,695 ha of community lands (e.g. paddy, oil palm smallholdings and rubber) may be lost to the PBH. Due to land tenure technicalities, many affected households may not get compensation for the loss of their homes and lands. The PBH will disproportionally impact Sabah’s Indigenous Peoples, with the Kadazandusun most affected. For this study to be constructive, we provide a low impact alternative alignment for a part of the PBH; discuss the socio-economic and cultural impacts of the PBH, and offer some perspectives on current planning procedures in Sabah to support more sustainable and equitable development.

## Introduction

It is estimated that human displacement caused in pursuit of economic development affects 15 million people annually [1]. This development-induced displacement occurs when people are forced to leave homes and lands due to the construction of: dams and irrigation; mining projects; commercial agricultural expansion; forestry and protected area establishment; construction of oil and gas pipelines; rail and road development; and, other types of large-infrastructure projects [2, 3]. Such types of development are beheld as essential steps towards economic growth and modernisation in many developing countries, but the outcome of such economic pursuit is often devastating for the displaced communities [4].

Road development, if well planned, can stimulate socio-economic growth at various scales [5, 6]. Roads can, importantly, promote accessibility of remote communities to essential services such as clinics, schools and markets, and they can also increase social mobility and migration, and therefore provide greater economic opportunities for peoples’ livelihoods and general wellbeing [6–8].

Road developments are expanding rapidly across the Earth, and it is estimated that by the year 2050 some 25 million kilometres of new roads will be paved [9]. However, roads, highways, rail and other linear infrastructure projects are often not well planned. Many road and highway projects have become controversial due to inadequate attention to social and environmental issues during planning and poor implementation [10, 11]. For Indigenous communities in remote areas, roads have, in some cases, decimated populations through the increased transmission of diseases [12, 13]. There have also been cases of the influx of migrants into newly opened areas due to road expansion, initiating land speculation [10]. Most notably, roads have also resulted in the compulsory acquisition of lands by the government [14].

The road network in Asia is expected to double within the next few years [15]. In Malaysia, between the years 2002 and 2018, the road network doubled by 177,858.4 km, with 75% (133,434 km) being paved roads [16, 17]. Under the Eleventh Malaysia Plan (2016–2020), a 5-year development plan aimed at achieving the 2020 Vision of the government, there was a further push for more roads, road upgrades to 4-lane highways and other infrastructure development projects [18]. One such project in the Eleventh Malaysian Plan was the Pan Borneo Highway (PBH), a federally funded project aimed at increasing connectivity within and between the two States of Sabah and Sarawak, located on the island of Borneo. At the State level for Sabah, the Sabah Development Corridor and the Sabah Structure Plan 2033, two overarching policy documents, aim to quadruple Sabah’s Gross Domestic Product between 2008 to 2025 through a number of high profile infrastructure development projects, one of which is the PBH [19, 20].

The idea of the PBH to connect Sabah and Sarawak stems from the 1960s, an initiative then known as the Trans-Borneo Highway, where 2-lane roads were constructed to link cities [21]. The revival of the PBH consists of three Phases comprising roughly 1,200 km in Sabah. Parts of Phase 1 (circa 706 km in length) are currently under construction i.e., the upgrading of the existing 2-lane roads to 4-lane dual carriage highways and a new 2-lane coastal highway in the north-west of Sabah [22]. The cost of the PBH was initially estimated at 16 billion Malaysian Ringgit (i.e., around USD3.85 billion) based on the Malaysian Highway Development Masterplan completed in 2008 [23, 24]. Although no empirical economic or traffic survey studies have ever been undertaken, prescriptive benefits stated by the Sabah government for the PBH range from: road safety [24]; the development of an industrial corridor; enhanced regional transportation connectivity; easier access to social services such as schools, markets, and hospitals; and, increased access to natural resources such as timber and minerals [20]. The PBH in Sabah is currently partially completed (in Phase 1), although behind schedule and over budget after a switch in contracting arrangements and government [25], and is subject to intense public debate, including around environmental and social impacts [26–29].

Currently, the government is clearing homes and lands for the PBH, often in the face of local disquiet and publicised human distress [30, 31]. The acquisition of alienated lands for public purposes, such as for infrastructures like the PBH, is through compulsory land acquisition under the Sabah Land Acquisition Ordinance (Sabah Cap. 69) [32]. Once the government gazettes its decision to acquire land, the land automatically vests in the government. Land owners, under this ordinance, only have the right to challenge the amount of financial compensation they will receive. For those that might have Native Customary Rights (NRC) that have not yet been recognised by the State, the government is not legally obligated to pay any compensation. Compensations or not, these households are plunged into situations of involuntary displacement and land and livelihood insecurity.

Unlike the environmental impacts of roads, assessments on the social impacts, especially on local and Indigenous communities has been inadequately quantified [33]. If fact, over the past decade, or more, there has been an expansive amount of literature assessing the impacts of roads and highways on wildlife, habitats and the environment. For example, studies have looked at the impacts of roads on bird and mammals including impacts on population abundance [11, 34, 35], as well as wildlife population tipping points [36], the way that animals use highway edges [37], and how behavioural changes can occur in species, such as the Asian and African elephant [38–40], tigers [41] and small mammals [42]. Other studies have looked at the impacts of road expansion on hunting and wildlife trade [43, 44], road kills [45], fire regimes [46], forested areas [47] and general environmental impacts [48, 49]. In Sabah, a study has already assessed the potential impacts of the Pan Borneo Highway on protected area connectivity, forest intactness and faunal dispersal, and provided mitigation opportunities within this context [50].

In this paper, we hope to contribute a robust methodology to the small but growing literature on the impacts of roads and highway development on communities, focusing on the Pan Borneo Highway in Sabah. We do this under three scenarios that range in the proposed highway corridor’s width to include estimates for a minimal 50m width, 75m width, and a maximum of 100m. In the three scenarios, we use high-resolution satellite imagery to estimate the number of homes that may be lost due to the PBH construction through compulsory acquisition by the government. In addition to the loss of homes is the loss of lands and livelihoods, and we estimate the extent of Native Titles that will need to be acquired by the government. We also look at the land uses associated with communities within these areas to understand better the impact that this highway may have on local and indigenous peoples’ livelihoods. Lastly, in order to explore the potential options for reducing displacement, and for this study to be constructive, we demonstrate a lower impact alternative route along part of the PBH as an example of how this highway could be constructed without displacing and impoverishing local communities.

## Materials and methods

### Pan Borneo Highway alignment

**Fig 1:**
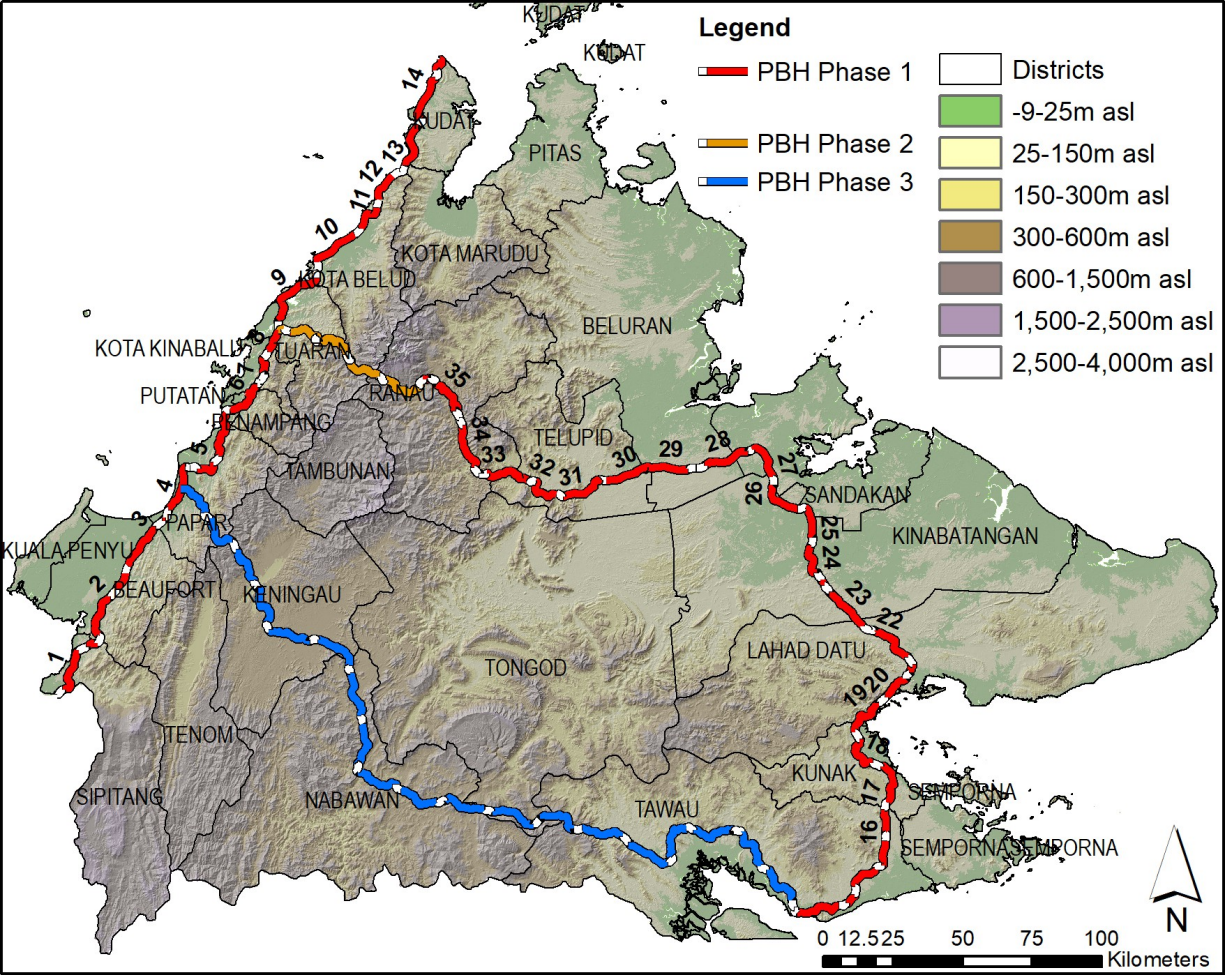
Pan Borneo Highway alignment. Alignment of the three phases of the Pan Borneo Highway (PBH) in Sabah. Phase 1 shown in red with work package numbers marked for the differing sections, as well as Phase 2 (in yellow), Phase 3 (in blue), and district boundaries and names, and elevation (in meters above sea level). Note, the alignments may change, dependent upon government decisions.

Images of the Sabah PBH were obtained from a number of sources to identify the planned alignments. These included maps from the Borneo Highway PDP Sdn Bhd website, Environmental Impact Assessment reports [51, 52], and the Sabah Structure Plan 2033 [20]. The available maps were georeferenced and the PBH alignments were digitised using ArcGIS 10.8.1, along with information on road names, work packages, development types (road upgrade or new road) etc. (Fig 1).

To ensure that the digitised alignments were as accurate as possible, the alignments were initially assessed and edited based on SPOT 5 high resolution (1.5 meters) imagery from years 2014–2015, just prior to the start of the PBH construction. Then, where Google Earth images were more recent than the SPOT 5 images, these were extracted, georeferenced and used to refine the PBH alignments. The digitised alignments followed existing 2–lane or gravel roads for the stretches involving the upgrading of existing roads. For the new highway sections, the digitised alignments followed the location obtained in various acquired (and georeferenced) Google Earth imagery, where evidence of the PBH construction could be seen (e.g. linear bulldozing for a highway).

To understand the potential width variations of the PBH, we obtained an official government shapefile for work package (WP) 6 (in Putatan, Penampang and Kota Kinabalu districts), which showed detailed information of the lands that will be developed for the PBH. This PBH shapefile included areas of the carriageway itself, paved and unpaved roadside areas, various drainage requirements, and areas demarcated for hill cutting and/or reinforcing slope stabilisation, and covered a length of 22.5 km. To understand the width variations along this 22.5 km alignment, we divided the length into intervals of 250 m and at each interval, we measured the total width in meters that would be required for clearing. From this, we then calculated the mean, median, and identified the minimum and maximum values. This information was important for providing realistic scenarios for the three widths we selected.

### Identifying potentially impacted villages, dwellings, and other buildings

To estimate the number of potential villages that the PBH may impact, we used a village point file layer that was initially downloaded from the Sabah Lands and Surveys Department’s online platform (http://www.jtuwma.net/), which was then verified and improved upon, where necessary.

To estimate the number of potentially affected houses (herein referred to as dwellings), and other building types that may be demolished due to the PBH construction, three width scenarios were used, which were 50m, 75m and 100m, based on our PBH width analysis described above. These widths included the 4-lane highway, a central reservation, and drainage on either side. The buffer shapefiles for these width scenarios were developed using the buffer tool in ArcGIS 10.8.1.

Using the three buffer width shapefiles, we then used high resolution (1.5 meters) SPOT 5 2014/2015 satellite imagery to initially identify buildings within the PBH buffers. These imagery were used as: (1) due to their high resolution they allowed for easy viewing of buildings, and later on for mapping land use types; and, (2) they were captured a year or two prior to the commencement of the construction of the PBH, which started in 2016. Meaning, these gave a snapshot (at an appropriate resolution) of the situation prior to the start of the implementation of the PBH. For mapping buildings, a building, in this case, is defined as a manmade structure for residential, social or commercial application. All buildings inside the buffers were digitised within a point shapefile (except for those identified as sheds). Where available, ‘street view’ images were used in Google Earth, to examine each ‘building’ to ensure that only buildings as defined above were digitised; and, to enable the identification of the buildings function i.e., dwelling, shop, workshop, school, mosque etc., which were recorded in the shapefile. For alignments where street view images were not available, we assigned the building type based on our best assessment of the building’s characteristics.

### Identifying community lands and livelihoods

To further understand the impacts on communities, we developed spatial data that reflected dominant community land use types within the 50m, 75m, and 100m PBH width scenarios. These land use types were: (1) wet paddy that included actively planted paddy and some areas of fallow or abandoned paddy; (2) identifiable vegetable agricultural smallholdings; (3) oil palm smallholdings; and (4) community mosaic areas that were composed of complex landscapes of diverse land uses that included dispersed homesteads and villages, smallholdings of various types such as rubber, coconut, hill rice, small-scale vegetable and fruit, small-scale food agriculture, areas of shifting-cultivation, orchards and small patches of forest, and additionally dispersed small patches of oil palm smallholdings. These land use type geo-informatic layers were developed from on-screen digitisation from SPOT 5 1.5 m satellite imagery for 2014/2015.

### Identifying dwellings and community lands on land titles

Additionally, we used cadastral data dated 23^rd^ January 2017, which was accessed from the Sabah Lands and Surveys Department online platform (www.jtuwma.net) on 2^nd^ February 2017. Within these cadastral data, there were over half a million land titles and five land title types: Town Lease (TL), Country Lease (CL), Provisional Lease (PL), Native Title (NT), and Field Register (FR). The first three types, TL, CL and PL are open to all citizens and non-citizens of Malaysia and are usually allocated for commercial development e.g. oil palm estates. In contrast, NT and FR are meant for Indigenous Peoples in Sabah and are often comprised of smallholdings and dwellings. We used these data to overlay with the identified dwellings and community lands within the three width scenarios of the PBH to identify the extent of land titles within the alignments.

### Identifying key Indigenous groups along the Pan Borneo Highway alignment

**Fig 2:**
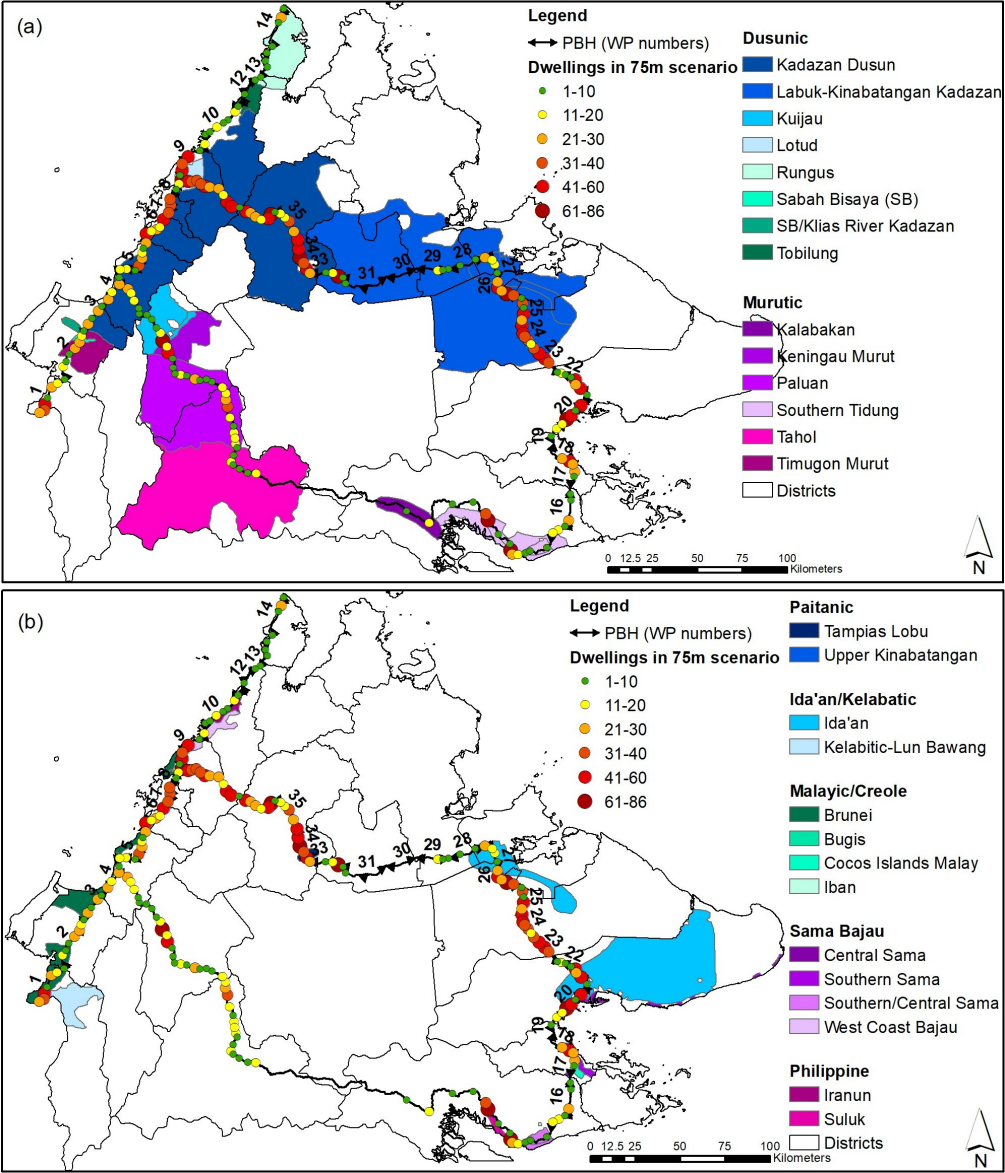
Impacted dwellings of the Pan Borneo Highway and traditional Indigenous territories. Maps showing the digitised alignment of the Pan Borneo Highway (PBH) (black line) in Sabah, along with the number of dwellings (coloured dots) that would be demolished within the 75m width scenario of the PBH; along with the geographic regions of the various Dusunic and Murutic languages (a), and Paitanic, Ida’an, Kelabatic, Malayic, Creole, Sama Bajau and Philippine languages (b), which indicate those ethnic groups that may be impacted by the PBH. The linguistic data is adapted and republished from Lewis et al. (2016) under a CC BY license, with permission from SIL International, original copyright (2016).

To understand which particular Indigenous groups the PBH construction may impact, we used existing language data from Lewis et al., [53]. These data were adapted and used by permission, © SIL International, (Ethnologue: Languages of Malaysia the Nineteenth edition data, 2016). The original map for Sabah identified 49 ethnic languages within five broad linguistic family groups, which was digitised and adapted to include relevant Indigenous groups for this study (Fig 2). We then overlaid the houses point file created for the dwelling analyses for each of the three highway width scenarios to identify the dominant ethnic groups along the PBH alignment.

### Identifying a lower impact alternative alignment for the Pan Borneo Highway

**Fig 3:**
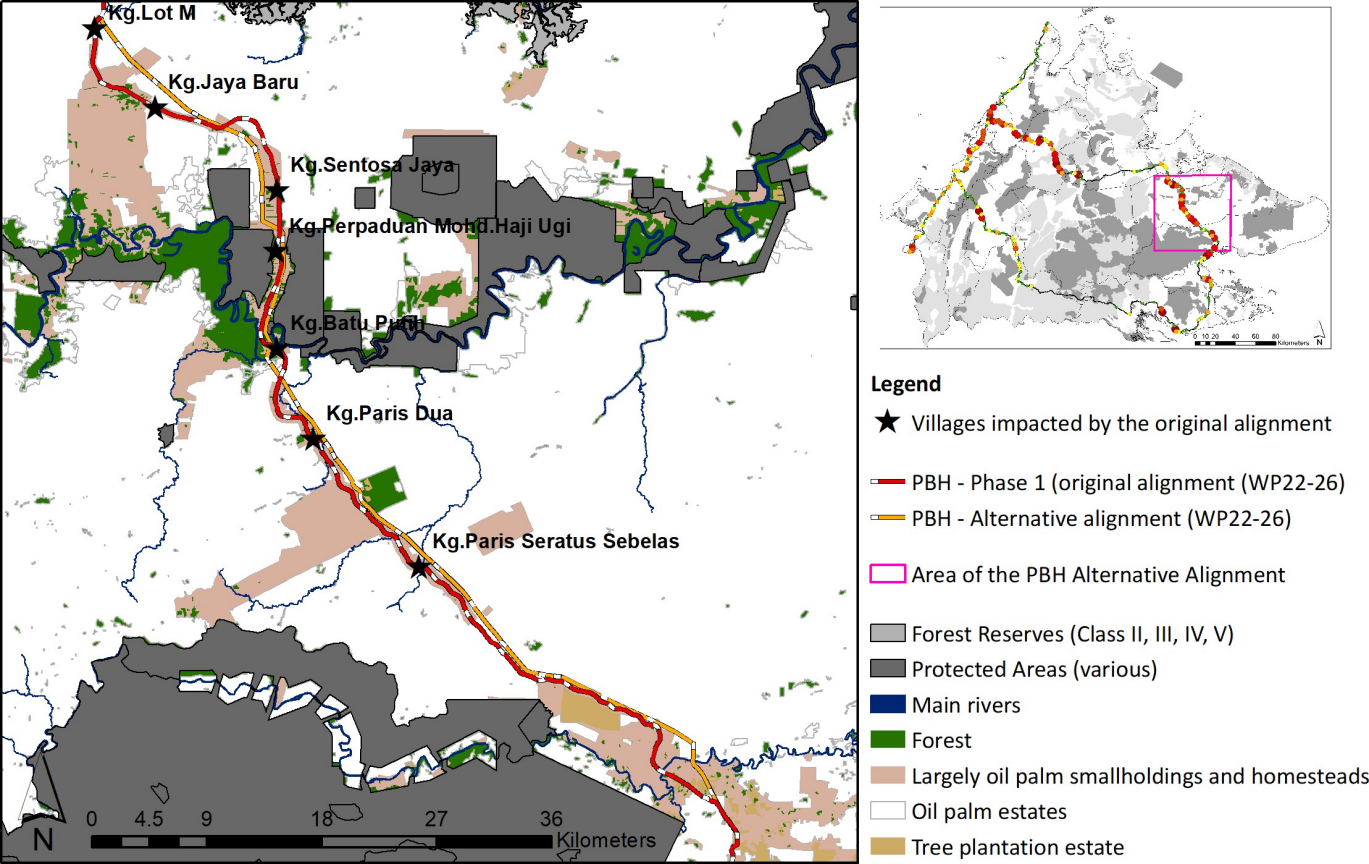
Alternative alignment for the Pan Borneo Highway. Top right map shows the area of the Pan Borneo Highway (PBH) alternative alignment within Sabah (pink box). The map on the left shows this area with the PBH’s original alignment (red and white line) and an alternative lower impact alignment (orange and white line) along with large areas of oil palm estates (white), protected areas/forest reserves (dark grey and light grey), unprotected forest (dark green), and community lands (largely Native titles) that contain homesteads and oil palm smallholdings (light pink) and village locations (yellow stars) and names.

To understand if there are alternative alignments that could prevent displacement of communities and still allow for the PBH to achieve its goals, we identified an alternative alignment for five work packages in Phase 1 (WP 22–26) in Lahad Datu and Kinabatangan districts (Fig 3). These work packages were based on our knowledge that construction had not yet begun, and that the surrounding landscape is relatively homogenous, meaning that there is a strong likelihood that an alternative lower impact route could be a viable solution engineering-wise.

To select an alternative route, we used the SPOT 5 high resolution (1.5 meters) satellite imagery to identify a viable alternative route that would avoid dwellings, as much as possible. To compare the current PBH alignment with our alternative alignment for these five work packages, we identified the impacted villages, the number of potentially destroyed dwellings, and the extent of possibly affected land use types within these two alignment scenarios.

## Results

### Pan Borneo Highway alignment

An estimated 1,227 km of the PBH alignment was identified and digitised, covering all three phases (Fig 1 and S1 Table). Phase 1 consisted of 35 work packages along 750 kilometres; and, Phases 2 and 3 spanned around 83 km and 394 km, respectively (Fig 1).

Based on the latest Google Earth images available at the time of data analysis (May 2020), clearing and road construction were observed in twelve work packages in Phase 1, spanning 164 km in length (S1 Table). Eight out of these 12 work packages were proposed to be road upgrades from 2-lanes to a 4-lane highway. However, from our observations within Google Earth, new roads (and not upgrading of 2-lane to 4-lane as per government plans) were identified for around 10.7 km in WP 1 (in Sipitang district), 13.6 km in WP 4 (Bongawan-Papar), and 14.8 km in WP 5 (Papar-Donggongon) (S1 Table). Additionally, for WP 5, 32.8 km of the alignment was identified and digitised, which was 21.1 km longer than the proposed length of 11.7 km [22]. For WP 4, the new road construction (c. 13.6 km) was likely an alternative alignment from that shown in the Sabah Structure Plan 2033 [20]. Ongoing clearing and construction were also observed in four proposed new highways, namely WP 6 (Putatan–Inanam), WP 7 (Inanam–Sepanggar), WP 10 (Pituru–Rampayan Laut) and WP 21 (Lahad Datu Bypass). Hill cutting occurred in WP 5 and WP 10.

The total distance of ongoing clearing and construction was approximately 165.1 km (c. 15% of all identified alignment for Phase 1), about 72.4 km of which involved road upgrade, whereas circa 92.7 km were land cleared for new roads. The shortest and the longest distance of cleared lands for the new roads were 6.2 km in WP 21 and 19.9 km in WP 5, respectively.

Minimum PBH widths of 50m and 60m were stated in government PBH documentation [54]. The actual width of the PBH, as calculated from the official shapefile data for WP 6, ranged from 50.5–306.8 m. The mean width was 84.8 m, and the median was 71.9 m, supporting the relevance of our selected width scenarios.

### Loss of dwellings and other buildings

We estimated that 65 villages in the 50m scenario will be impacted by the PBH, with this increasing to 80 villages for the 75m width, and 93 villages for the 100m width scenario.

For dwellings, 1,712 were identified within the 50m width scenario, 4,372 for the 75m scenario (Fig 2), and 7,093 for the 100m scenario (Table 1). Phase 1 is the longest phase and is currently in construction, and will cause the majority of losses of people’s dwellings (Table 1).

**Table 1:** Building numbers that may be impacted by the Pan Borneo Highway.

Phase	Buildings	Numbers within road widths of:		
		50m	75m	100m
All phases (1,127 km)	Number of dwellings	1,712 (80%)	4,372 (81%)	7,093 (76%)
	Number of other buildings	431 (20%)	1,058 (19%)	2,186 (24%)
	Total number of identified buildings	2,143	5,430	9,279
Phase 1 (750 km)	Number of dwellings	1,233 (87%)	3,006 (84%)	4,851 (77%)
	Number of other buildings	181 (13%)	586 (16%)	1,427 (23%)
	Total number of identified buildings	1,414	3,592	6,278
Phase 2 (83 km)	Number of dwellings	292 (74%)	628 (76%)	867 (72%)
	Number of other buildings	102 (26%)	203 (24%)	330 (28%)
	Total number of identified buildings	394	831	1,197
Phase 3 (394 km)	Number of dwellings	187 (56%)	738 (73%)	1,375 (76%)
	Number of other buildings	148 (44%)	269 (27%)	429 (24%)
	Total number of identified buildings	335	1,007	1,804

Summary table for the number of dwellings and other buildings that may be demolished due to the construction of the Pan Borneo Highway (PBH), with data broken down into its three phases and highway width scenarios of 50m, 75m and 100m wide.

In regards to Sabah’s Indigenous Peoples, the traditional territories of 27 different Indigenous groups were identified along the PBH. The Kadazandusun Peoples may be the most impacted ethnic group with 24.6–37.7% of all dwellings, across the three width scenarios, were identified within their traditional lands (Table 2 and Fig 2). The Labuk-Kinabatangan Kadazan people may be the second most impacted group, with 15.1–19% of dwellings identified within their traditional lands, then the Lotud with around 6–7% of dwellings in the PBH path (Table 2 and Fig 2). Details on other potentially impacted groups can be seen in Table 2.

**Table 2:** Number and % of dwellings per Indigenous group within the three width scenarios.

Family groupings of languages	Dominant languages	Number and (%) of dwellings within PBH width scenarios of:		
		50m	75m	100m
Dusunic	Kadazandusun	593 (37.7%)	1,118 (28%)	1,575 (24.6%)
	Labuk-Kinabatangan Kadazan	238 (15.1%)	758 (19%)	1,113 (17.4%)
	Lotud	100 (6.3%)	278 (7%)	447 (7%)
	Rungus	40 (2.5%)	67 (1.7%)	80 (1.3%)
	Kuijau	12 (0.8%)	39 (1%)	81 (1.3%)
	Sabah Bisaya & Klias River Kadazan	2 (0.1%)	3 (0.1%)	5 (0.1%)
	Tobilung	2 (0.1%)	4 (0.1%)	6 (0.1%)
Murutic	Paluan	66 (4.2%)	250 (6.3%)	456 (7.1%)
	Timugon Murut	19 (1.2%)	39 (1%)	82 (1.3%)
	Tahol	18 (1.1%)	52(1.3%)	86 (1.3%)
	Keningau Murut	17 (1.1%)	73 (1.8%)	135 (2.1%)
	Kalabakan	12 (0.8%)	19 (0.5%)	25 (0.4%)
	Southern Tidung	5 (0.3%)	26 (0.7%)	65 (1%)
Malayic	Brunei	72 (4.6%)	198 (5%)	355 (5.6%)
	Iban	11 (0.7%)	40 (1%)	57 (0.9%)
Paitanic	Tampias Lobu	52 (3.3%)	79 (2%)	98 (1.5%)
Sama Bajau	West Coast Bajau	47 (3%)	75 (1.9%)	134 (2.1%)
	Southern Sama	38 (2.4%)	174 (4.4%)	276 (4.3%)
	Central Sama	16 (1%)	63 (1.6%)	102 (1.6%)
Ida’an	Ida’an	38 (2.4%)	95 (2.4%)	172 (2.7%)
Danao	Iranun	27 (1.7%)	41 (1%)	70 (1.1%)
Kelabitic	Lun Bawang	15 (1%)	62 (1.6%)	102 (1.6%)
Creole	Cocos Islands Malay	1 (0.1%)	5 (0.1%)	9 (0.1%)
*Multiple dominant languages*				
Dusunic/Ida’an	Labuk-Kinabatangan Kadazan / Ida’an	47 (3%)	91 (2.3%)	121 (1.9%)
Sama Bajau / Bisayan	Southern Sama / Suluk	28 (1.8%)	59 (1.5%)	109 (1.7%)
Paitanic/Ida’an	Upper Kinabatangan / Ida’an	18 (1.1%)	40 (1%)	74 (1.2%)
Dusunic/Murutic	Kuijau / Keningau Murut	13 (0.8%)	47 (1.2%)	107 (1.7%)
Bisayan/Murutic/Malayic	Suluk / Southern Tidung / Bugis	9 (0.6%)	88 (2.2%)	204 (3.2%)
Bisayan/Murutic	Suluk / Southern Tidung	8 (0.5%)	46 (1.2%)	90 (1.4%)
Malayic/Sama Bajau	Brunei / West Coast Bajau	7 (0.4%)	17 (0.4%)	31 (0.5%)
Dusunic / Murutic	Sabah Bisaya / Timugon Murut	3 (0.2%)	24 (0.6%)	56 (0.9%)
Murutic / Sama Bajau	Southern Tidung / Southern Sama & Central Sama	1 (0.1%)	16 (0.4%)	47 (0.7%)
Bisayan / Sama Bajau / Murutic	Suluk /Southern Sama&Central Sama /Southern Tidung	-	10 (0.3%)	26 (0.4%)
-	Area with multiple languages	76 (4.8%)	157 (3.96%)	297 (4.6%)
Unknown	n/a	61 (3.9%)	219 (5.5%)	400 (6.3%)
**TOTAL**		**1,712**	**4,372**	**7,093**

The number (and percentage) of houses identified within the three width scenarios of the Pan Borneo Highway as per their association with traditional territories of Indigenous groups, identified by language. Language spatial data sourced from Lewis et al. (2016).

For the other (non-dwelling) buildings, we identified 431 (within 25 differing types) in the 50m scenario, 1,058 (in 36 different types) for the 75m width scenario, and 2,186 (in 44 different types) for the 100m width scenario (S2 Table).

### Loss of community lands and livelihoods

Whilst there are many other types of land use impacted by the PBH construction, communities are using around half the lands affected. We identified 3,420 ha of community-used lands within the 50m width scenario, 5,074 ha in the 75m width scenario, and 6,695 ha within the 100m width scenario (Table 3). The community lands that would likely be converted to the PBH include: 144–303 ha of paddy fields; 67–134 ha of identifiable vegetable smallholdings (largely grown in a particular region of Sabah); 945–1,898 ha of oil palm smallholdings; and, 2,264–4,360 ha of community mosaic areas that include dwellings, rubber, small-scale plantings of hill rice and vegetables, fruit trees and even the odd plot of oil palm (Table 3).

**Table 3:** Extents of community used lands within the Pan Borneo Highway path.

Land uses	Extent in hectares (ha)		
	50m	75m	100m
Wet paddy	144 ha	223 ha	303 ha
Vegetable agriculture	67 ha	100 ha	134 ha
Oil palm smallholdings	945 ha	1,419 ha	1,898 ha
Community mosaic areas	2,264 ha	3,332 ha	4,360 ha
**TOTAL EXTENT**	**3,420 ha**	**5,074 ha**	**6,695 ha**

Calculations of the extent in hectares of community used lands that will be converted to the Pan Borneo Highway (PBH) under three width scenarios of 50m, 75m and 100m.

### Loss of dwellings and community lands on titles and with no titles

Available data on government issued land titles suggests that 726 ha of Native Titles/Field Register titles may be obtained by the government through compulsory acquisition for the 50m PBH width scenario, 1,290 ha for the 75m width, and 1,918 ha for the 100m width scenario (Table 4). Based on the number of unique land titles identified, this could affect around 3,673 to 5,070 households (Table 4). The number of hectares and the number of affected titles for other land title types are shown in Table 4.

**Table 4:** Land title types that may be impacted by the Pan Borneo Highway.

Titles	Total extent in ha (number of titles)		
	50m	75m	100m
Native Title (NT)	678 ha (3,514)	1,212 ha (4,342)	1,809 ha (4,839)
Field Register (FR)	48 ha (159)	78 ha (188)	109 ha (231)
Country Land (CL)	617 ha (1,954)	1,277 ha (2,615)	2,039 ha (3,089)
Provisional Lease (PL)	159 ha (81)	246 ha (96)	335 ha (1,080)
Town Land (TL)	4 ha (136)	10 ha (211)	17 ha (237)
Unmarked titles	2,488 ha (1,738)	3,155 ha (2,156)	3,667 ha (2,356)
Protected Areas	472 ha	708 ha	947 ha
Production Forest Reserves	377 ha	567 ha	758 ha
Areas with no data (possibly State land)	1,299 ha	1,988 ha	2,664 ha
**TOTAL EXTENT**	**6.151 ha**	**9,241 ha**	**12,345 ha**

Calculations of the combined extent in hectares of land title types (and the number of potential land titles impacted) that the government may acquire for the construction of the Pan Borneo Highway (PBH).

We also identified unmarked titles that were demarcated but had no information as to their title type. These totalled 2,488 ha, 3,155 ha, and 3,667 ha for the 50m, 75m, and 100m width scenarios, respectively. Additionally, we identified large areas that had no titles and were not associated with any protected areas or forest reserves. These areas are assumed to still be State lands (i.e., 1,299 ha were identified for the 50m scenario, 1,988 ha for the 75m scenario, and 2,664 ha for the 100m width scenario) (Table 4).

We found that 23–28% of dwellings, identified across the three width scenarios, were on Native Title/Field Register, though this could be higher as we also found 31–26% of dwellings (across the three width scenarios) on titles that lacked title type information (Table 5). We identified 8–16% of dwellings, across the three width scenarios, were on commercial titles (Country Land or Provisional Title), and the remaining dwellings, from 29–38%, across the three width scenarios) were in locations with no land titles, assumed to be State land (Table 5).

**Table 5:** Dwelling and community lands on land titles that may be impacted by the Pan Borneo Highway.

	Number (and %) of dwellings, and extent (and %) of community lands in the three PBH width scenarios		
	50m	75m	100m
No. dwellings on Native Title/Field Register	397 (23%)	1,133 (26%)	2,015 (28%)
No. dwellings on Country Land/Provisional Title	137 (8%)	545 (12%)	1,112 (16%)
No. dwellings on Town Lands	3 (<1%)	35 (1%)	74 (1%)
No. dwellings on Unknown title types	526 (31%)	1,235 (28%)	1,818 (26%)
No. dwellings potentially on State lands	649 (38%)	1,424 (33%)	2,074 (29%)
***Total no*. *dwellings***	**1,712**	**4,372**	**7,093**
Community lands on Native Title/Field Register	513 ha (15%)	927 ha (18%)	1,392 ha (21%)
Community lands on Country Land/Provisional Title	238 ha (7%)	480 ha (9%)	750 ha (11%)
Community lands on Town Lands	1 ha (<1%)	1 ha (<1%)	2 ha (<1%)
Community lands on Unknown title types	1,357 ha (40%)	1,767 ha (35%)	2,102 ha (31%)
Community lands on potentially State lands	1,311 ha (38%)	1,901 ha (37%)	2,449 ha (37%)
***Total extent of Community Lands***	**3,420 ha**	**5,076 ha**	**6,695 ha**

Summary of the number and proportion (%) of dwellings, and total hectares (ha) of lands and their proportions (%) within Native Title/Field register titles; Country Land/Provisional Lease; Town Lands; titles that had been demarcated but had no code; and, possibly on State lands, for the three Pan Borneo Highway (PBH) width scenarios of 50m, 75m and 100m.

For the community lands, 15–21% of were on Native Title/Field Register titles. This may be higher as for 40% (in the 50m scenario) to 31% (in the 100m scenario) of lands lacked sufficient data on titles types (Table 5). From 7–11% of community lands were on Country Land/Provisional Lease Titles; and in the region of 37% were possibly on State lands (Table 5).

### Wider impacts of the Pan Borneo Highway

Three sites where the PBH cuts through large areas of wet paddy were identified. These were in the districts of Sipitang (though 125 ha of wet paddy), Papar (225 ha), and Kota Belud (7,949 ha).

### Lower impact alternative alignment

Compared to the government’s original alignment, our alternative alignment for the five work packages (WPs 22–26) was 7.94 km shorter in length (Table 6 and Fig 3). Because of the positioning of the alternative alignment that runs largely at the back of community areas, the number of impacted villages and dwellings would be far less (Table 7). Under our alternative alignment, for the 75m width scenario, only 18 dwellings would be lost versus 832 under the government’s current alignment (Table 7). For lands, under the 75m width scenario, the alternative alignment would also use far less lands associated with communities (i.e.,228 ha), than that in the original alignment (567 ha), as it would cut through more oil palm estates (Table 8).

**Table 6:** Comparison of the Pan Borneo Highways original and alternative alignment lengths.

Work package (WP)	Original alignment length (km)	Alternative alignment length (km)	Difference between the two alignments (km)
WP22	22.26 km	21.41 km	0.85 km
WP23	26.38 km	25.21 km	1.17 km
WP24	15.91 km	13.94 km	1.97 km
WP25	14.64 km	13.66 km	0.98 km
WP26	18.06 km	15.09 km	2.97 km
TOTAL LENGTH	97.25 km	89.31 km	7.94 km

Lengths of the original Pan Borneo Highway (PBH) alignment and the proposed lower impact alternative alignment for Work Packages 22 to 26.

**Table 7:** Comparison of impacted dwellings of the Pan Borneo Highways original and alternative alignment.

	No. dwellings in 50m width	No. dwellings in 75m width	No. dwellings in 100m width
PBH original length	247	832	1,281
PBH alternative alignment	11	18	59
Difference between the two alignments	236	814	1,222

Comparison of the alignments of the Pan Borneo Highway (PBH) with the number of dwellings identified within the three width scenarios (of 50m, 75m and 100m) that span from Work Package 22 to 26.

**Table 8:** Land use types and extents within the original Pan Borneo Highway alignment and in the alternative alignment.

Land uses	Extent in hectares (ha)		
	50m	75m	100m
*PBH original alignment*			
Community lands (oil palm smallholdings/homesteads)	380 ha	567 ha	754 ha
Oil palm estate	101 ha	153 ha	205 ha
Degraded areas/forest	0.8 ha	2.2 ha	4 ha
Agroforestry estate/other	5.1 ha	7.8 ha	10.4 ha
*PBH alternative alignment*			
Community lands (oil palm smallholdings/homesteads)	152 ha	228 ha	302 ha
Oil palm estate	268 ha	402 ha	538 ha
Degraded areas/forest	18.8 ha	28 ha	37.8 ha
Agroforestry estate/other	8.3 ha	12.3 ha	16.1 ha

Calculations of the extents (in hectares) of land uses identified within the original Pan Borneo Highway (PBH) alignment for work packaged 22 to 26, compared to land uses identified within the alternative alignment, under three width scenarios of 50m, 75m and 100m.

## Discussion

If roads are well planned, they have the potential to greatly serve society by stimulating socio-economic growth and enhancing accessibility of communities to essential services such as clinics and schools, and enable better access to markets and employment opportunities [55]. However, if inadequately planned, such roads can negatively affect the very communities these roads aim to serve [10].

### The loss of dwellings for Indigenous and local communities

We estimated 1,712–7,093 dwellings in 65–93 villages that lie within the PBH path. The most likely width scenario, of 75m, may see 4,382 dwellings in around 80 villages lost in the pursuit of constructing this 4-lane highway (Fig 2 and Table 1). The high number of dwellings in close proximity to the road is likely a result of when Sabah’s rural road network was established in the decades after World War Two. Many communities relocated from their historic riverine settlements in remote areas where land was in short supply to live along new roads [56, 57]. This pattern of (ribbon) development resulted in land titles being granted along roadsides. As a result, the widening of the current 2-lane road to the 4-lane PBH, will cause the displacement of many families and impact many villages.

Whilst, some of these dwellings belong to non-Indigenous people, the most recent government census data shows continued fidelity of Sabah’s main Indigenous groups within their traditional lands [58]. Using spatial data on Indigenous groups, we found that the PBH will disproportionately impact a few ethnic groups (Table 2). The Kadazandusun people (Sabah’s largest ethnic group) will likely be the most impacted group, then the Labuk-Kinabatangan Kadazan Peoples (Orang Sungai), then the Peoples of the Lotud Paluan and Brunei groups (Fig 2 and Table 2).

### Loss of lands and livelihoods for Indigenous communities

For many Indigenous People in Sabah, especially those in remote areas, there is great dependency on land for food, household income, and cultural identity [59]. We identified 3,420–6,695 ha of community-used lands (i.e., paddy, mixed-community areas and oil palm smallholdings) within the PBH alignment scenarios that may be at risk (Table 3). The loss of these lands will impact these communities in a number of ways.

#### The PBH impact on culturally important wet paddy

We identified 144–303 ha of wet paddy fields within the PBH alignment (Table 3). Wet paddy provides essential food security, which is especially important if household income is low or in times of social disruption e.g. in times of a pandemic [60]. Yet, for some, the connection to the practice of paddy planting is far greater. For certain Indigenous groups, wet paddy carries sacred and cultural significance. The Kadazandusun are traditionally farmers of paddy, planting varieties of wet rice (*parai dumoh* in local tongue), and hill rice (*parai tidong*) [61]. In fact, paddy plays a vital role in the spiritual and cultural heritage of the Kadazandusun, who traditionally believe in the rice spirit (*Bambarayon*) and celebrate a rice harvest festival (*Tadau Kaamatan)* [62]. Published evidence shows that government policies have heavily eroded the Kadazandusun culture and traditions [63, 64]; and that the construction of the PBH (through hundreds of hectares of paddy in Kadazandusun areas) will likely further erode cultural identity, as well as impact households economy and food security.

The PBH may, however, have wider implications for paddy. For example, the building of roads and highways may disrupt local hydrology and threaten irrigation systems needed for rice cultivation, as well as create issues of sedimentation that can dry up paddy fields and clog up waterways [65, 66]. Paddy may also be impacted by pollution, especially heavy metals that are often associated with highway construction, and pose risk to people’s health [67–70]. We identified three locations where the PBH alignment cuts through significant areas of wet paddy. These were in the districts of Sipitan (125 ha total), Papar (225 ha), and Kota Belud (7,949 ha). In Papar, at least 80 hectares of paddy have already suffered from irrigation disruption due to an already constructed section of the PBH [71]. Such disruptions are often due to planning of road location without consideration of local land use; the use of substantial embankments to prevent road flooding, which disrupt water flows with culverts and bridges being built with limited hydrological data about farmers’ needs; and, little if any local consultation [65].

For Kota Belud, the construction of the PBH is underway, cutting through the largest area of paddy in Sabah. Being the largest area of paddy in the state, this region has seen significant government investment to increase Sabah’s rice self-sufficiency. The State invests RM 113 million (USD 28 million) in rice production in Sabah annually [72]; and the Federal Government allocated a further RM 381 million (USD 94 million) for Sabah’s rice production, under the Eleventh Malaysian Plan (2016–2020) [73, 74]. This is the same plan that proposed the PBH, meaning the implementation of one federally funded project (i.e., the multi-billion PBH) may create significant issues for another, with thousands of hectares of paddy at risk.

#### PBH impacts on other traditional and economic crops

We also identified 2,264–4,360 ha of community mosaic areas (Table 3), consisting of homesteads, swidden agriculture, hill rice, fruit and vegetable crops, orchards, patches of forest and mixed agroforestry and rubber. These areas likely represent significant family investments, providing subsistence and small-scale livelihood streams, as well as intangible values to residents.

Hill paddy, like wet paddy, is also culturally significant for Kadazandusun and Murut communities. Equally, it is important for subsistence, like many of the other fruit and vegetable products grown by Indigenous communities. Such subsistence agriculture is still widely practised in Sabah and continues to be important in sustaining many Indigenous livelihoods and household wellbeing. Examples of such communities in areas where the PBH is planned, include many Kadazandusun and Murut villages in the western coastal lands, the northern district of Kudat and within Sabah’s interior [59]. Hill paddy, has also been documented at contributing around RM 180 (USD 45) per month to farmers’ household income [75, 76].

The main economic crop within the community mosaic areas, however, is rubber. According to one study, just under half of Sabah’s rubber smallholders earn between RM 1,001–2,000 (USD 248–495) per month, and just over half making RM1,000 (USD 248) or less [77]. How significant this is becomes clear when compared with the mean household income for Indigenous Sabahans, which is estimated at around RM 3,752 (USD 902) per month [78]. Such land-based incomes are particularly important in more remote areas that the PBH will traverse, where lower household incomes are the norm e.g. RM 1,500 (USD 371), RM800 (USD 198), RM300 (USD 74) per month [79–82].

We also identified 945–1,898 ha of oil palm smallholdings within the PBH alignment, largely in the lowlands of the eastern districts and Telupid (Table 3). For example, one study for Sabah and Sarawak showed that one-third of smallholders earned between RM 1,000–2,500 (USD 248–619) per month, and a quarter under RM 1,000 (USD 248) per month [83]. Another study on oil palm smallholders in four districts (that the PBH will cut through i.e., Telupid, Tongod, Beluran and Kinabatangan), showed that oil palm smallholdings contributed 33–50% of household’s income, making RM 1,500 (USD 371) per month on average [79]. The loss of monthly incomes from oil palm smallholdings would, therefore, be significant for many households.

These assessments of land use and livelihoods of communities along the PBH alignment indicate that such communities are dependent on their land assets for food and income, as well as residence. Furthermore, with the added economic impact of the Covid-19 pandemic, displacement for these households may collapse them into dire poverty, especially for those who are unable to secure waged labor opportunities elsewhere.

### Issues surrounding land laws and compensation

Villagers displaced by the PBH will need to find new houses and/or lands for relocation. The capacity to do this is heavily dependent on funds derived from government compensation. Yet, not every displaced person is entitled to receive compensation and will depend on the occupant’s status.

Firstly, owners of private (or alienated) land, who have legally recognised titles (e.g. Native Title, Country Lease) that are acquired for the construction of the PBH, will likely be eligible for compensation under the Sabah Land Acquisition Ordinance (Cap. 69). The amount of compensation is calculated based on the current market value of the land, which includes buildings, planted trees or standing crops [84]. We identified that 23–28% of dwellings (across the three width scenarios) and 15–21% of community lands, were within Field Register/Native Titles, and owners should, bylaw, receive compensation (Table 5). These estimates may be higher, as 26–31% of the identified dwellings and 31–40% of community lands were within titles that lacked title type data, with a proportion of the likely also being Field Register/Native titles enabling occupants to receive compensation (Table 5).

The second category of occupants are Native People who occupy state land and assert legitimate claims to Native Customary Rights (NCR), or *adat*, to those areas [85, 86]. NCR are not explicitly defined in the Land Acquisition Ordinance, however, they are defined in the Land Ordinance (see S1 Appendix), and, the definition of “*land*” within the Land Acquisition Ordinance encompasses property of every tenure or description, which, arguably, includes claims of NCR [32, 87]. If NCR claims by the occupants of such state land are legally recognised by government, compensation for the loss of assets on these lands should be paid. However, successful NCR claims are fairly rare as the definition, under law, is a highly modified form to which many traditional practises and systems do not adhere [88, 89]. Further, the legal process of claiming NCR is typically arduous and long, landing many communities in land disputes for decades [90, 91]. As a result, occupant’s rights to compensation for the loss of land and assets in areas with claims of NCR is certainly questionable.

The third category comprises of occupants who, by law, illegally occupy state lands. These people do not hold any title deeds to the land, nor do they have any valid claims of NCR, and as a result are not entitled to any compensation under the Land Acquisition Ordinance. These illegal occupants may comprise an array of people. Unfortunately, Sabah has many stateless and undocumented people. A significant proportion of undocumented people include Indigenous People to Sabah, whose parents/grandparents failed to register themselves after independence in 1963 [92]. These stateless Indigenous Peoples, are ineligible to basic services (education, healthcare, legal employment) and have no legal rights to land [92]. We found 29–38% of dwellings and around 37% of community lands were possibly on State land, and may demonstrate areas used by stateless Indigenous People (Table 5). Further to this, many of the road reserve titles in the cadastral dataset did not have title type information, and could make up a substantial proportion of the ‘unknown title’ types for dwellings and/or community lands (Tables 4 and 5). In fact, this was illustrated in a documentary series that interviewed residents evicted by the government because of the PBH [31]. In these cases, dwellings and lands illegally used, such as in road reserves, may be considered ‘illegal’ and occupants may not be entitled to any compensation.

We do not have data on actual compensation made to land occupants. Government may, in some cases, give some compensation to all affected people, regardless of the occupant’s status out of concern for social welfare, or simply to reduce occupants’ resistance to forced eviction. As a result, we cannot conclude whether fair compensation has been offered, and whether payments have been made prior to eviction. However, based on a handful of known cases, it seems that compensation has been problematic, for those offered, with accusations of compensation being low and delayed. For example, villagers from Kampung Tanaki, and nearby settlements in Penampang, affected by WP6 of the PBH, protested over delays in compensation and the allegedly manipulated valuation of land compensation that was calculated at only RM 7 (USD 1.7) per square foot, an insufficient amount to acquire alternative land in the area [93]. Timeliness matter as many displaced families lack the assets to build alternative homes and livelihoods quickly and therefore can face long periods of uncertainty and potential homelessness. Interviews with some affected residents along the PBH, stated that their eviction letter gave them 14 days to leave their home, leaving little time to find living solutions [31]. For those people who may not receive any compensation at all, the impact of the PBH will be greatly felt and will likely marginalise these communities even further.

### Other socio-economic impacts of the PBH

It is not only those who lose their dwellings and lands who will be impacted, a wider community could also feel the burden of the PBH. A village that loses residents may experience damage to its local economy, which is important for school, energy, health, retail and other government and commercial services. Road to highway upgrading may also be accompanied by a loss of roadside income-generating activities, especially informal road-side stalls that are important for the livelihoods of many roadside villages throughout Sabah [94].

Furthermore, the legacy of ribbon development will mean that many villages will span both sides of the 4-lane high speed highway. Mobility wise, it will become dangerous for children, elderly, and the wider community members to socialise and carry out daily activities. Villages not directly affected may also be impacted by having to house or support displaced community members (e.g. parents, grandparents, village elders) from other villages. All these have psychological underpinnings towards wellbeing.

Indeed, the emotional trauma of being ousted from one’s home and lands will likely be considerable for many. Displacement may scatter kinship groups and families, weakening culture and social networks and systems [95]. This may be felt more by Indigenous communities, and especially by the elderly, women and children [96, 97]. Long-term studies of development-related displacement have demonstrated increases in landlessness, joblessness, homelessness, marginalisation, food insecurity, substance abuse, increased morbidity and mortality, loss of access to common property, and social disintegration, even across generations [4].

Although not discussed in depth here, the array of more well known environmental impacts that result from road building such as the impacts on wildlife, forests and various ecosystem services [11, 50], could further exacerbate the impacts felt by local communities [15]. For example, the PBH in Sabah will cut through two Bornean elephant range areas (in Telupid and Kalabakan, a paper on this is forthcoming, in addition to a paper on High Conservation Value Areas and the impacts of highways). For Telupid, the PBH will cut through a protected area and 27 km of elephant range and it is feared that this will have negative consequences to the local villagers safety, their properties and smallholdings, as well as general safety to road users in this section of the highway [98]. Another example, is the potential impacts of the PBH on numerous mangrove patches in north-west Sabah, which will likely cause hydrological disturbances, in addition to general degradation, forest loss and pollution [99]. These environmental impacts will likely reduce the livelihoods of fishermen and overall food security of local fisher communities within this region [100].

### Proposed lower impact alignment

Given the potential magnitude of the impacts of the PBH, finding alternative lower impact routes could be an important way forward. Our alternative alignment for five of the 35 PBH work packages in Phase I (i.e., WP 22–26) would largely follow electric pylon tracks, traversing the back-end of community lands and through oil palm estates (Table 8 and Fig 3). This would be instead of widening the existing road, which is fringed by villages and homesteads. Not only was our alternative alignment shorter (by 7.94km; Table 6), but at the 75m width scenario would impact just 18 dwellings, as opposed to 832 dwellings under the current government plan, and would impact 228 ha of community lands versus 567 ha identified within the original alignment (Tables 7 and 8 and Fig 3). By re-routing WPs 22–26, communities could remain *in-situ* and benefit from having the existing road (now relieved of dangerous through-traffic), and an accessible highway.

Finding alternative alignments is still possible for the PBH. For example, in Telupid, communities mobilised to pressure the former government to re-route part of the PBH to avoid displacement of their village [29]. The government accepted this request, however, the alignment was subsequently moved south through a protected area and an elephant migration, mentioned above [98]. Due to the potentially disastrous environmental impacts associated with this re-route, a counter alternative was provided by NGOs that would avoid protected areas and most of the elephant range [26, 98]. The government is currently considering the counter alternative alignment. Whilst this example is complicated, it does demonstrate the ability of government to change alignments (if there is will to do so), and that there are often various alternatives that can be considered in order to minimise environmental and social impacts.

Within our mapping, we also identified areas where ‘new’ routes for the 4-land PBH were under construction that were originally meant to be an up-grade of the existing 2-lane road (i.e., 10.7km, 1, 13.6km and 14.8km, in WP 1, 4 and 5, respectively) (S1 Table). The underlying motivation for re-aligning these sections of the PBH remains unclear, and could be cost related. Nevertheless, due to the location of these sections they will impact less villages and displace fewer communities than the original alignment.

In addition to rerouting some of the work packages of the PBH, certain sections could remain 2-lane roads, especially in areas of environmental sensitivity and to avoid further forest fragmentation [50]. Excess funds, could then better serve the Sabah population by upgrading some of the thousands of kilometres of gravel and earth roads that keep many hundreds of villages isolated with little access to basic services and opportunities. Indeed, largescale displacement doesn’t have to be synonymous with road development and such projects can, and should, aim to benefits to the masses, including rural, marginalised communities.

### An opportunity to improve processes and procedures

Within the lifespan of this federally funded project, there have been three changes in government. These changes have delayed implementation [101], creating opportunities for the current government to re-consider PBH alignments in the light of fuller information on social and environmental impact. Conducting Social Impact Assessments (SIA) could now broaden the information available to the government planners. More significantly, Special Environmental Impact Assessments (SEIA), could still be undertaken; these are aimed at major projects that have impact beyond individual work package and project sites and on the socio-economic and cultural welfare of local communities [102]. SEIAs require more comprehensive information than regular Environmental Impact Assessments (EIAs), and public hearings are employed to identify issues and recommendations [103]. Had SEIAs been undertaken for the PBH they might have helped the government to identify and address the kinds of issues addressed in this paper.

Government, researchers, civil society and community/Indigenous organisations are improving the quality and availability of digital spatial information. There is an opportunity for sharing data and knowledge to improve early-stage planning of such projects to avoid unnecessary impacts and costs. In fact, there is potential to transform Sabah’s infrastructure sector as a whole. Sabah has already initiated such transformation in its palm oil sector, by committing to certified sustainable palm oil under a Roundtable on Sustainable Palm Oil’s (RSPO) Jurisdictional Certification approach. To do this, complex assessments and processes are needed and are being rolled out under a Jurisdictional Certification Steering Committee that comprises government, civil society and private sector. The infrastructure sector could follow suit, elevating their planning processes and procedures (to put minimising social and environmental impacts at the forefront), and governing the planning and implementation of such projects under a multi-stakeholder committee to facilitate transparency, sustainability, and equality in large infrastructure projects. Such an approach would address one of the major limitations on current Environmental Impact Assessments. Namely that they are too late in the process to be used to guide optimal routing options, and that they often present the environment as just an obstacle to implementation, rather than seeing the environment as a valuable asset. Transforming the infrastructure sector, would be timely as Sabah aims to construct more highways, extensive railways, oil and gas pipelines, dams, and expand its mining [20], all of which could see further displacement of communities, and the loss of biodiversity and ecosystem services upon which communities depend [104–107].

But it is not too late and the current government has the opportunity to change how it plans and rolls out these transformative mega-projects. We hope this study has provided insights on the potential cumulative impacts faced by communities from the PBH. We have shown that low impact alternatives are possible and hope these can be advanced through open consultative planning with NGOs and impacted communities.

We hope that this study provides a sound methodology for quantifying large-scale socio-economic impacts from infrastructure development projects, especially for Indigenous Peoples and local communities. These methods are important for governments, consultants, researchers, and NGOs, in order to help transform how infrastructure development planning is conducted, globally, to reduce the displacement of communities and the negative consequences that can follow road development. Further, there is an increasing need for road impact studies to integrate socio-economic data with biodiversity and environmental data so that we can further understand the real impacts of such projects on people and the planet.

## Supporting information

S1 Table Pan Borneo Highway work packages and details of construction. (DOCX)

S2 Table Various building types that may be impacted by the Pan Borneo Highway. (DOCX)

S1 Appendix Customary rights definition. (DOCX)
