# Supplementary material for: The socio-economic and cultural impacts of the Pan Borneo Highway on Indigenous and local communities in Sabah, Malaysian Borneo
Source: PLoS One. 2022 Jun 27;17(6):e0269890. doi: 10.1371/journal.pone.0269890 (PMC9236263; doi:10.1371/journal.pone.0269890)
Supplement: S1 Table — (DOCX) [file pone.0269890.s001.docx]

**S1 Table. Pan Borneo Highway work packages and details of construction.**

**The socio-economic and cultural impacts of the Pan Borneo Highway on Indigenous and local communities in Sabah, Malaysian Borneo**

**S1 Table:** The phases, regions, road names, associated work packages, proposed development, actual development and the current (ongoing) development progress of the Pan Borneo Highway alignments.

| Phase | Region/Road name | Work package (WP) | Proposed development (No. of lanes) | Actual development (upgrade 2-lane to 4-lane and/or new highway) | Length of cleared areas for ‘New’ road despite being proposed as ‘Upgrade’ | Potential length (km) | Progress | Max. width of clearing/construction (m) |
| --- | --- | --- | --- | --- | --- | --- | --- | --- |
| 1 | Southwest: Sindumin–Donggongon | 1: Sindumin–Kg.Melalia | Upgrade (4) | Upgrade & New | 10.7 km | 28 | On-going | 260.4 |
|  |  | 2: Kg.Melalia–Beaufort | Upgrade (4) | Upgrade |  | 31 | On-going | 105.9 |
|  |  | 3: Beaufort–Bongawan | Upgrade (4) | Upgrade |  | 26 | On-going | 66.4 |
|  |  | 4: Bongawan–Papar | Upgrade (4) | Upgrade & New | 13.6 km | 23 | On-going | 93.0 |
|  |  | 5: Papar–Donggongon**** | Upgrade (4) | Upgrade & New | 14.8 km | 32.8 | On-going | 167.8 |
|  | KK Outer Ringroad (KKOR) | 6: Putatan–Inanam | New (4) | New |  | 23 | On-going | 260.9 |
|  |  | 7: Inanam–Sepanggar | New (4) | New |  | 10 | On-going | 211.9 |
|  |  | 8: Sepanggar–Bulatan Berungis | Upgrade (4) | Na (Note: Already 4 lanes in 2014) |  | 33 | Not started | Na |
|  | Northwest: Serusup–Tg. Simpang Mengayau* | 9: Serusup–Pituru | New (2) | Na |  | 11 | Not started | Na |
|  |  | 10: Pituru–Rampayan Laut | New (2) | New |  | 32 | On-going | 219.3 |
|  |  | 11: Rampayan Laut–Sarang | New (2) | Na |  | 6 | Not started | Na |
|  |  | 12: Sarang–Temuno | New (2) | Na |  | 14 | Not started | Na |
|  |  | 13: Temuno–Bingolon | New (2) | Na |  | 20 | Not started | Na |
|  |  | 14: Bingolon–Tg. Simpang Mengayau | New (2) | Na |  | 29 | Not started | Na |
|  | Southeast: Tawau–Lahad Datu Bypass | 15: Tawau–Semporna | Upgrade (4) | Upgrade |  | 6 | On-going | 98.8 |
|  |  | 16: Tawau Airport–Sg. Kalumpang | Upgrade (4) | Upgrade |  | 33 | On-going | 81.7 |
|  |  | 17: Sg. Kalumpang–Madai | Upgrade (4) | Na |  | 20 | Not started | Na |
|  |  | 18: Madai–IGN Estate | Upgrade (4) | Na |  | 19 | Not started | Na |
|  |  | 19: IGN Estate–Agri Harvest | Upgrade (4) | Na |  | 21 | Not started | Na |
|  |  | 20: Agri Harvest–Sepagaya | Upgrade (4) | Na |  | 14 | Not started | Na |
|  |  | 21: Lahad Datu Bypass | New (4) | New |  | 6.2 | On-going | 158.4 |
|  | Northeast: Lahad Datu Bypass–Mile 32, Sandakan | 22: Lahad Datu Bypass–Kg.Sandau | Upgrade (4) | Na |  | 22 | Not started | Na |
|  |  | 23: Kg.Sandau–Sg. Takala | Upgrade (4) | Na |  | 26 | Not started | Na |
|  |  | 24: Sg. Takala–Kg.Perpaduan Dtk Moh | Upgrade (4) | Na |  | 16 | Not started | Na |
|  |  | 25: Kg.Perpaduan Dtk Moh–Sukau | Upgrade (4) | Na |  | 15 | Not started | Na |
|  |  | 26: Sukau–Kg.Lot M | Upgrade (4) | Na |  | 18 | Not started | Na |
|  |  | 27: Kg.Lot M–Mile 32, Sandakan | Upgrade (4) | Upgrade |  | 18 | On-going | 84.2 |
|  | Central: Mile 32, Sandakan–Ranau* | 28: Mile 32, Sandakan–Moynod | Upgrade (4) | Na |  | 22 | Not started | Na |
|  |  | 29: Moynod–Sapi Nangoh | Upgrade (4) | Na |  | 19 | Not started | Na |
|  |  | 30: Sapi Nangoh–Sg.Baoto | Upgrade (4) | Na |  | 18 | Not started | Na |
|  |  | 31: Sg.Baoto–Telupid | Upgrade (4) | Na |  | 19 | Not started | Na |
|  |  | 32: Telupid–Kg.Lumou Baru | Upgrade (4) | Na |  | 19 | Not started | Na |
|  |  | 33: Kg.Lumou Baru–Kg.Toupos | Upgrade (4) | Na |  | 20 | Not started | Na |
|  |  | 34: Kg.Toupos–Kg.Nabutan | Upgrade (4) | Na |  | 24 | Not started | Na |
|  |  | 35: Kg.Nabutan–Ranau | Upgrade (4) | Na |  | 27 | Not started | Na |
| 2 | Tamparuli–Ranau | Na | Upgrade (4) | Na |  | 83 | Not started | Na |
| 3 | Kimanis–Keningau–Kalabakan–Tawau | Na | Upgrade (4) | Na |  | 394 | Not started | Na |

Na = Not available

* = Lack of latest and clear Google Earth images to track the actual development progress.

** = Part of Phase 1, yet information are conflicting among the proposals.

*** = Unclear whether this is the actual Papar (bypass) stretch as shown in SSP2033.

****= Conflicting information on the extent of WP5.
