# Supplementary material for: The socio-economic and cultural impacts of the Pan Borneo Highway on Indigenous and local communities in Sabah, Malaysian Borneo
Source: PLoS One. 2022 Jun 27;17(6):e0269890. doi: 10.1371/journal.pone.0269890 (PMC9236263; doi:10.1371/journal.pone.0269890)
Supplement: S2 Table — (DOCX) [file pone.0269890.s002.docx]

**S2 Table. Various building types that may be impacted by the Pan Borneo Highway.**

**The socio-economic and cultural impacts of the Pan Borneo Highway on Indigenous and local communities in Sabah, Malaysian Borneo**

**S2 Table:** Types and numbers of potentially affected buildings identified within a 50 meter, 75 meter and a 100 meter width of the Pan Borneo Highway.

|  | **Numbers within road widths of:** | | |
| --- | --- | --- | --- |
| **Types of buildings** | **50m** | **75m** | **100m** |
| Houses | 1712 | 4372 | 7093 |
| Bird Nest House | 1 | 1 | 3 |
| Church | 2 | 9 | 33 |
| Clinic | 1 | 2 | 8 |
| Community Hall | 5 | 16 | 28 |
| Factory | 8 | 36 | 119 |
| Farm | 2 | 3 | 8 |
| Flat | 0 | 0 | 7 |
| Futsal Court | 0 | 0 | 1 |
| Gas Station | 1 | 11 | 33 |
| Guardhouse | 4 | 10 | 16 |
| Homestay | 1 | 2 | 3 |
| Hospital | 0 | 0 | 3 |
| Hotel | 3 | 16 | 27 |
| Information Centre | 0 | 1 | 1 |
| Internet Centre | 0 | 1 | 4 |
| Kindergarten | 1 | 7 | 10 |
| Library | 1 | 2 | 2 |
| Lodge | 1 | 6 | 14 |
| Market | 0 | 0 | 1 |
| Milk Collecting Centre | 0 | 1 | 1 |
| Mosque | 6 | 18 | 43 |
| Nursery | 3 | 5 | 6 |
| Office | 12 | 53 | 114 |
| Old Folks Home | 0 | 0 | 1 |
| Police Station | 0 | 1 | 5 |
| Post Office | 0 | 1 | 1 |
| Power Station | 1 | 1 | 5 |
| Public Toilet | 3 | 4 | 4 |
| Pump Station | 0 | 2 | 2 |
| Quarantine Station | 0 | 1 | 1 |
| Recycle Centre | 0 | 1 | 1 |
| Resort | 8 | 13 | 27 |
| School | 9 | 41 | 125 |
| Shop | 323 | 704 | 1318 |
| Shop/Flat | 0 | 0 | 31 |
| Storage | 1 | 2 | 3 |
| Temple | 0 | 1 | 3 |
| Training Centre | 3 | 3 | 5 |
| Tv Transposer Station | 0 | 1 | 1 |
| Warehouse | 0 | 2 | 2 |
| Water Filter Plant | 0 | 0 | 1 |
| Water Pump | 1 | 7 | 8 |
| Water Treatment Plant | 0 | 0 | 4 |
| Workshop | 30 | 73 | 153 |
| **Total** | **2143** | **5430** | **9279** |
