# Supplementary material for: The socio-economic and cultural impacts of the Pan Borneo Highway on Indigenous and local communities in Sabah, Malaysian Borneo
Source: PLoS One. 2022 Jun 27;17(6):e0269890. doi: 10.1371/journal.pone.0269890 (PMC9236263; doi:10.1371/journal.pone.0269890)
Supplement: S1 Appendix — (DOCX) [file pone.0269890.s003.docx]

**S1 Appendix. Customary Rights definition.**

**The socio-economic and cultural impacts of the Pan Borneo Highway on Indigenous and local communities in Sabah, Malaysian Borneo**


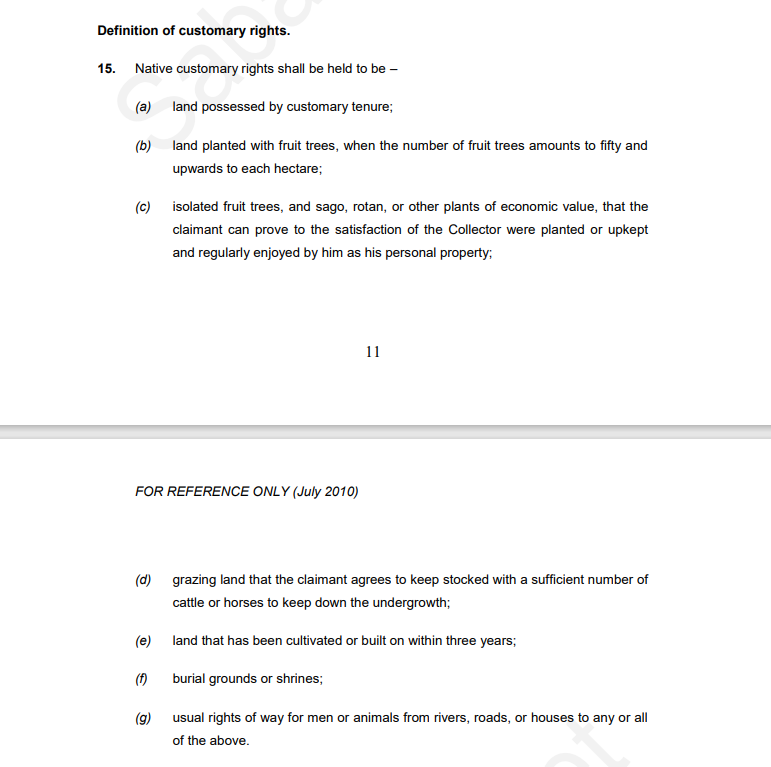


**S1 Fig:** Extract taken from Pages 11 and 12 of the Sabah Land Ordinance (Cap 68., Section 15) that states the Definition of Customary Rights.
